# A steroid like phytochemical Antcin M is an anti-aging reagent that eliminates hyperglycemia-accelerated premature senescence in dermal fibroblasts by direct activation of Nrf2 and SIRT-1

**DOI:** 10.18632/oncotarget.11229

**Published:** 2016-08-11

**Authors:** Kumar K.J. Senthil, Vani M. Gokila, Jeng-Leun Mau, Chin-Chung Lin, Fang-Hua Chu, Chia-Cheng Wei, Vivian Hsiu-Chuan Liao, Sheng-Yang Wang

**Affiliations:** ^1^ Department of Forestry, National Chung Hsing University, Taichung, Taiwan; ^2^ National Chung Hsing University/University of California at Davis, Plant and Food Biotechnology Center, National Chung Hsing University, Taichung, Taiwan; ^3^ Department of Food Science and Biotechnology, National Chung Hsing University, Taichung, Taiwan; ^4^ Taiwan Leader Biotech Company, Taipei, Taiwan; ^5^ School of Forestry and Resource Conservation, National Taiwan University, Taipei, Taiwan; ^6^ Department of Bioenvironmental Systems Engineering, National Taiwan University, Taipei, Taiwan; ^7^ Agricultural Biotechnology Research Center, Academia Sinica, Taipei, Taiwan

**Keywords:** Antcin M, antrodia salmonea, hyperglycemia, stress-induced premature senescence, SIRT-1, Gerotarget

## Abstract

The present study revealed the anti-aging properties of antcin M (ANM) and elucidated the molecular mechanism underlying the effects. We found that exposure of human normal dermal fibroblasts (HNDFs) to high-glucose (HG, 30 mM) for 3 days, accelerated G_0_/G_1_ phase arrest and senescence. Indeed, co-treatment with ANM (10 μM) significantly attenuated HG-induced growth arrest and promoted cell proliferation. Further molecular analysis revealed that ANM blocked the HG-induced reduction in G_1_-S transition regulatory proteins such as cyclin D, cyclin E, CDK4, CDK6, CDK2 and protein retinoblastoma (pRb). In addition, treatment with ANM eliminated HG-induced reactive oxygen species (ROS) through the induction of anti-oxidant genes, HO-1 and NQO-1 *via* transcriptional activation of Nrf2. Moreover, treatment with ANM abolished HG-induced SIPS as evidenced by reduced senescence-associated β-galactosidase (SA-β-gal) activity. This effect was further confirmed by reduction in senescence-associated marker proteins including, p21^CIP1^, p16^INK4A^, and p53/FoxO1 acetylation. Also, the HG-induced decline in aging-related marker protein SMP30 was rescued by ANM. Furthermore, treatment with ANM increased SIRT-1 expression, and prevented SIRT-1 depletion. This protection was consistent with inhibition of SIRT-1 phosphorylation at Ser47 followed by blocking its upstream kinases, p38 MAPK and JNK/SAPK. Further analysis revealed that ANM partially protected HG-induced senescence in SIRT-1 silenced cells. A similar effect was also observed in Nrf2 silenced cells. However, a complete loss of protection was observed in both Nrf2 and SIRT-1 knockdown cells suggesting that both induction of Nrf2-mediated anti-oxidant defense and SIRT-1-mediated deacetylation activity contribute to the anti-aging properties of ANM *in vitro*. Result of *in vivo* studies shows that ANM-treated *C. elegens* exhibits an increased survival rate during HG-induced oxidative stress insult. Furthermore, ANM significantly extended the life span of *C. elegans*. Taken together, our results suggest the potential application of ANM in age-related diseases or as a preventive reagent against aging process.

## INTRODUCTION

Prematureskin agingis caused by several factors, including intense physical and psychological stress, alcohol intake, poor nutrition, environmental pollution, UV exposure and diabetes [1]. Hyperglycemia is a characteristic feature of diabetes mellitus (DM), and the clinical involvement of skin in diabetic complications such as impaired wound healing, foot ulceration, and premature skin aging are well studied [2, 3]. The proliferative capacity of skin fibroblasts harvested from diabetic subjects is reduced, as they have reduced replicative life span [4]. Likewise, human skin fibroblasts harvested from normal donors cultured in hyperglycemic medium result in reduction in the population doubling required to reach replicative senescence [5]. These findings suggest that accelerated cellular senescence resembling premature aging are also complications of diabetes.

Previous studies have shown that replicative senescence of human diploid fibroblasts (HDFs) or melanocytes is caused by the exhaustion of their proliferative potential [1]. Any proliferative cell types such as endothelial cells, lung cells, retinal pigment epithelial cells, melanocytes and skin fibroblasts undergo stress-induced premature senescence (SIPS) *in vitro* when exposed to sub-cytotoxic concentrations of oxidative-stress stimuli such as hydrogen peroxide (H_2_O_2_), hypoxia (*p*O2), *tert*-butylhydroperoxide (*tert*-BHP), ultraviolet radiation (UV) and hyperglycemia [6]. Over the last two decades several studies have been conducted to elucidate the cellular and molecular mechanisms of SIPS in skin fibroblasts, and have identified oxidative stress as playing a crucial role in the development of SIPS [7]. Increasing oxidative stress is frequently associated with aging and age-related disorders [8]. Reactive oxygen species (ROS) act as signaling molecules, whereas increased levels are damaging for DNA, proteins, and lipids as well as detrimental to cellular functions. Under normal physiological conditions, cells are equipped with an anti-oxidant defense system to eliminate pro-oxidants, but this system fails with over production of ROS [9]. Substantial evidence indicates that hyperglycemia- [10] and hydrogen peroxide [11]-induced ROS generation promotes cellular senescence and growth arrest, thus resulting in SIPS in fibroblasts. Accordingly, prevention of hyperglycemia-associated dermal fibroblast senescence may be a potential target to arrest the development of premature skin aging.

Many dietary components exert beneficial effects on the aging process, such as polyphenols, flavonoids, terpenoids, vitamins and omega-3-fatty acids [12]. These components exert anti-oxidant effects not only by scavenging free radicals but also by modu­lating signal transduction pathways such as *de novo* expression of antioxidant genes including hemoxygenase-1 (HO-1), NAD(P)H: quinone oxidoreductase-1 (NQO-1), glutathione-*S*-transferase (GST), γ-glutamylcestine synthetase (γ-GCLC), and superoxide dismutase (SOD) [13]. Transcriptional activation of antioxidants or detoxifying genes is predominantly regulated by a redox-sensitive transcription factor NF-E2 related factor-2 (Nrf2). Both *in vitro* and *in vivo* studies suggest that dietary phytochemicals are able to activate Nrf2 signaling thereby ameliorating the anti-oxidant defense system [13].

Accumulating evidence suggests that the activation of silent mating type information regulation 2 homologs (sirtuins), a family of NAD^+^-dependent class III histone deacetylases, extends life span and promotes longevity and healthy aging. In particular, sirtuin-1 (SIRT-1), a mammalian ortholog of yeast SIRT-2 plays a functional role in human aging by means of deacetylation, a protein activity that plays a crucial role in cellular senescence, such as p53, FoxO1 and E2F1 [14, 15]. A previous study demonstrated that hyperphosphorylation of SIRT-1 at serine 47 (S47) by mitogen-activated protein kinases (MAPKs) resulted SIRT-1 depletion and increased cellular senescence [16].

*Antrodia cinnamomea* is a precious medicinal mushroom that has long been used as a traditional Chinese medicine for the treatment of liver diseases, food and drug intoxication, diarrhea, abdominal pain, hypertension, allergies, skin itching and tumorigenic diseases [17]. *A. cinnamonea* is one of the richest sources of unique compounds such as antcins, anticinates, antrodins and antroquinonls [18]. Our recent study has shown that the chemical fingerprints of *A. cinnamomea* and its relative species *A. salmonea* are mostly identical. However, a few compounds including antcin M (ANM) and methyl anticinate K were only identified in *A. salmonea* [17]. Antcins, steroid-like compounds, exhibited various biological effects such as anti-oxidant, anti-inflammation, anti-cancer and cardioprotection. Previously, we reported that antcin C protects human hepatic cells from oxidative injury through the activation of Nrf2-dependent anti-oxidant genes [19]. However, the other effects of these potentially beneficial compounds have not been investigated. In this study, we screened a potent anti-aging compound from a group of antcins and investigated the effects of ANM on SIPS in HNDFs by analyzing changes in the expression of the above mentioned proteins. The effect of ANM was compared with known agents *N*-acetylcysteine and resveratrol for their anti-oxidant and SIRT-1 activation, respectively.

## RESULTS

### High-glucose accelerates growth arrest and senescence in HNDFs through the induction of ROS

**Figure 1:**
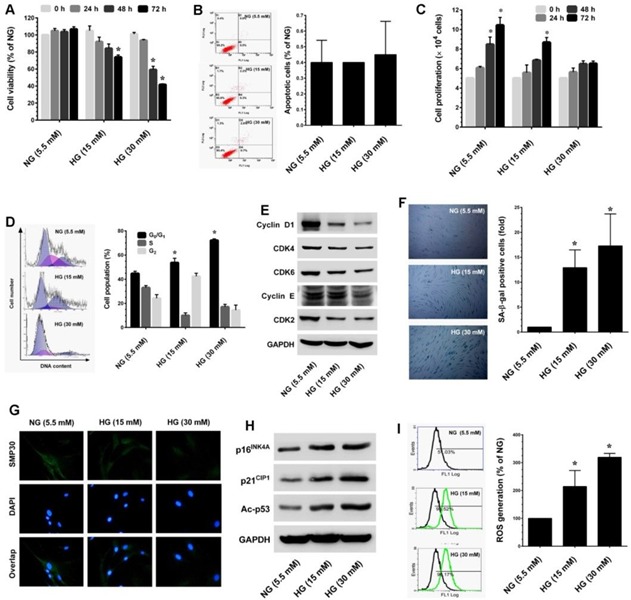
High-glucose (HG) accelarates stess-induced premature senescence in dermal fiboblasts **A.** HNDFs were incubated with increasing concentrations of HG (15 and 30 mM) for 24-72 h. Cell viability was measured by MTT assay. The percentage of viable cells was compared with normal glucose (NG, 5.5 mM). **B.** Apoptosis was determined by Annexin-V/PI staining. Percentages of apoptotic cells are shown in the histogram. **C.** To determine cell proliferation, HNDFs 5 × 10^4^ cells/well in a 6-well plate were incubated with HG for 72 h. Number of viable cells was quantified by the tryphan blue exclusion method using a hemocytometer. **D.** Cell-cycle distribution was measured by flow cytometer using propidium iodide (PI). Percentage of cell population are shown in the histogram. **E.** Immunoblotting was performed to determine the expression levels of cell-cycle regulatory proteins including cyclin D1, cyclin E, CDK4, CDK6 and CDK2. GAPDH served as an internal control. **F.** Cellular senescence was determined by senescence-associated β-galactosidase (SA-β-gal) assay as desceribed in Materials and Methods. The left panel shows representative figures and the right panel shows quantitative analysis of SA-β-gal positive cells per microscopic field. **G.** Immunofluroscence analysis shows expression and localization of SMP30 in HG-treated HNDFs. **H.** Western blot analysis shows the protein expression levels of p16^INK4A^, p21^CIP1^, and p53 acetylation in HG-induced HNDFs. GAPDH served as an internal control. **I.** The intracellular ROS level was dertermined by flow cytometry using DCH-DA flurogenic probe. Left panel shows representative figures and the right pane shows quantitative analysis of intracellular ROS in HG-treated HNDFs. Results are expressed as mean ± S.E.M of three indipendent expriments. Statistical significance was set at *P < 0.05 compared to NG *vs.* HG.

Oxidative stress is one of the major factors that plays a key role in the onset of senescence. Hyperglycemia-induced oxidative stress-mediated senescence has been well-studied in human vascular endothelial cells [20]. However, very few studies have investigated this phenomenon in other cell systems. Therefore, to establish a human dermal fibroblast senescence model, we utilized an established oxidative stress-mediated senescence model, which involved incubating cells with high glucose (> 30 mM) for 72 h [21]. To determine the cytotoxic effect of HG on the human dermal fibroblast-derived cell line CCD966SK, cells were incubated with increasing doses of HG (5.5, 15 and 30 mM) for 24-72 h and the cell viability was measured by MTT assay. Exposure to HG caused a dose- and time-dependent reduction in cell number. Particularly, treatment with high dose (30 mM HG) for 72 h reduced cell number to 41.7% (Figure 1A). Next, to examine whether the reduction in cell number was associated with apoptotic cell death, apoptosis was determined by Annexin-V/PI staining. Results of flow cytometric analysis showed that there was no significant increase in apoptotic-positive cells in HG treatment groups when compared to the control (NG) group (Figure 1B). Therefore, we hypothesize that HG may induce growth arrest/senescence in fibroblasts, which may be the reason for the reduction in cell number. Proliferation assay shows that HG caused dose- and time-dependent growth arrest in HNDFs (Figure 1C). Indeed, after treatment with HG (15 and 30 mM) for 72 h there was sustained proliferation, which is equal to the initial seeding. To further clarify these results, cell-cycle analysis was performed. Treatment with HG arrested HNDFs in the G_1_-S transition phase as evidenced by increased cell population in the G_0_/G_1_ phase from 46.1% (NG) to 51.1% and 73.1% by 15 and 30 mM of HG, respectively (Figure 1D). We further examined cell-cycle progression by quantifying cyclins and cyclin-dependent kinase (CDK) expression levels in HG-induced HNDFs. Results from immunoblotting strongly support the above observation that G_1_-S transition regulatory proteins such as cyclin D1, CDK4, CDK6, cyclin E and CDK2 were significantly down-regulated by HG in a dose-dependent manner compared with cells that had been cultured for the same time course in NG (Figure 1E). Accumulation of cells in the G_0_/G_1_ phase is one of the characteristic features of senescence. Therefore, we sought to examine whether HG induces senescence in HNDFs utilizing a senescence-associated β-galactosidase (SA-β-gal) assay. As we expected, an increased number of SA-β-gal positive cells were observed in HG-treated cells and this increase was noted in a dose-dependent manner (Figure 1F). Loss of senescence marker protein-30 (SMP30) expression is frequently observed in senescent cells [22]. In the present study, we also found that endogenous expression of SMP30 was significantly reduced by HG in a dose-dependent manner (Figure 1G). In addition, western blot analysis further supported our observation that senescence-associated modulation in proteins including p16^INK4A^, p21^CIP1^ and acetylation of p53 were significantly increased by HG (Figure 1H). HG increased intracellular reactive oxygen species (ROS), a major event triggering senescence, cell-cycle arrest and apoptosis in a variety of human cells. To determine whether the HG-induced growth arrest and senescence were mediated by ROS, the intracellular ROS levels were measured by flow-cytometry using a DCF-DA probe. As shown in Figure 1I, treatment with 15 and 30 mM HG increased mean fluorescence intensity to nearly 3-fold of the oxidation-dependent fluorogen DCF-DA, which is proportional to the increase in intracellular ROS. Taken together, these data confirm that HG can cause growth arrest and senescence in HNDFs without inducing cell death. Moreover, HG-induced ROS generation may play a crucial role in the onset of growth arrest and senescence in HNDFs. Furthermore, this is the first report indicating hyperglycemia-induced oxidative stress-mediated senescence in HNDFs.

### Screening of anti-aging substances from *A. cinnamomea* and *A. salmonea*

**Figure 2:**
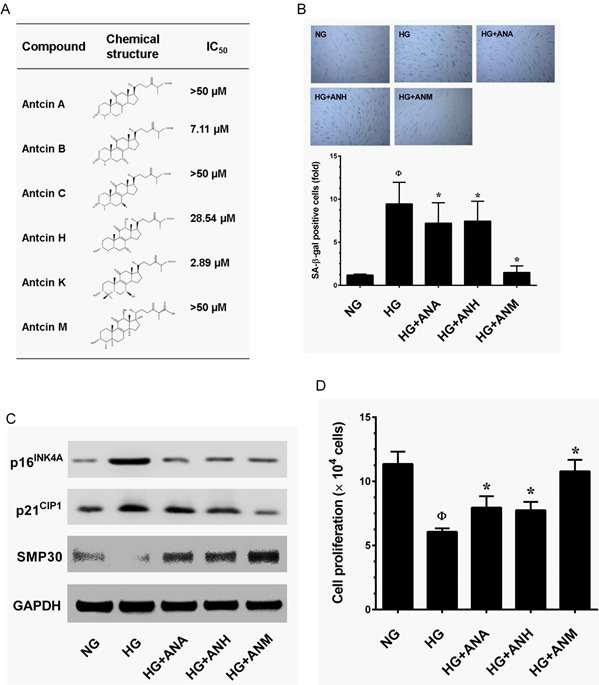
Effects of antcins on HG-induced senescence in HNDFs **A.** Type of antcins subjected to screen for potential anti-aging agents for skin aging. Names and chemical structures of the antcins are shown in the left and centrer column, respectively. The cytotoxic effects of antcins against HNDFs. Briefly, HNDFs were incubated with increasing concentrations (1, 5, 10 and 20 μM) of antcins including antcin A, antcin B, antcin C, antcin H, anticn K and antcin M for 72 h and the cell viability was determined by MTT assay. The 50% inhibitory concentrations (IC_50_) of each compound against HNDFs are shown in right column. **B.** Cells were incubated with antcin A (10 μM), antcin H (10 μM) and antcin M (10 μM) in the presence of HG (30 mM) for 72 h. Cellular senescence was determined by SA-β-gal activity. The left panel shows representative figures and the right panel shows quantitative analysis of SA-β-gal positive cells per microscopic field. **C.** HNDFs were incubated with antcin A (10 μM), antcin H (10 μM) and antcin M (10 μM) in the presence of HG (30 mM) for 72 h. Senescence-associated marker proteins such as p16^INK4A^, p21^CIP1^ and SMP30 were determined by western blot analysis. **D.** To determine the effect of antcins on cell proliferation efficacy, HNDFs 5 × 10^4^ cells/well in 6-well plates were incubated with antcins in the presence of HG (30 mM) for 72 h. Number of viable cells were quantified by the tryphan blue exclusion method. Results are expressed as mean ± S.E.M of three indipendent expriments. Statistical significance was et at ^Ф^*P* < 0.05 compared to NG *vs* HG and *P < 0.05 compared to HG *vs.* samples.

Anticins are ergostane-type triterpenoids that have been reported to be anti-oxidant, anti-inflammatory and anti-cancer agents. However, other pharmacological properties of antcins have not been studied. In this study we screened an anti-aging agent from a group of antcins including antcin A (ANA), antcin B (ANB), antcin C (ANC), antcin H (ANH), antcin K (ANK) and antcin M (ANM). Prior to the investigation, the cytotoxicity of antcins against HNDFs was determined. As shown in Figure 2A, ANB and ANK exhibited strong cytotoxicity to HNDFs with an IC_50_ value of 7.11 and 2.89 μM, respectively. However, ANA, ANC, ANH and ANM did not show significant cytotoxicity to HNDFs up to the high treatment concentration (20 μM) and the IC_50_ values were >50 μM (Figure 2A). Next, we examined the protective effects of antcins on HG-induced HNDF senescence, cells were co-incubated with HG and antcins (ANA, ANH and ANM) for 72 h, senescence was measured by SA-β-gal assay. Treatment with ANM showed significant protection against HG-induced HNDF senescence as evidenced by reduction in a number of SA-β-gal positive cells from 9.59-fold to 1.51-fold, whereas ANA and ANH showed moderate inhibition as SA-β-gal positive cells were reduced to 7.46-fold and 8.13-fold, respectively (Figure 2B). In addition, results from immunoblotting analysis confirmed that HG-induced upregulation of senescence-associated proteins such as p16^INK4A^ and p21^CIP1^ were significantly downregulated by ANM, whereas ANA and ANH showed a moderate inhibition, which is concomitant with the result of SA-β-gal assay (Figure 2C). Moreover, compared with ANA or ANH, ANM rescued HG-induced SMP30 depletion and significantly upregulated in HNDFs (Figure 2C). To further clarify, the effect of antcins, HG-induced reduction in cell proliferation was determined. As shown in Figure 2D, a two-fold increase in cell proliferation was observed in the ANM treatment group, whereas ANA and ANH partially increased cell proliferation compared to the HG treatment group. These data showed that out of the antcin group, ANM is a potent anti-aging component. Therefore, next we explored the molecular mechanism underlying the protective effect of ANM.

### Antcin M blocked HG-induced growth arrest in HNDFs

**Figure 3:**
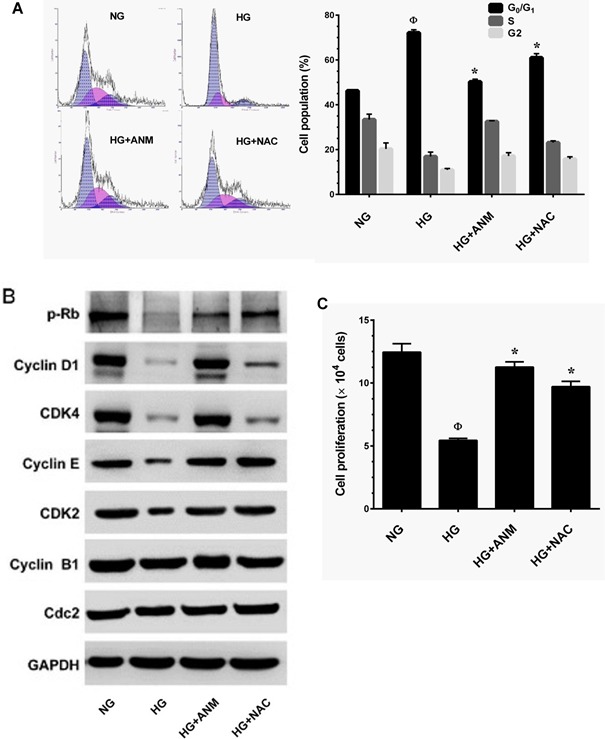
Antcin M blocked HG-induced growth arrest in HNDFs **A.** HNDFs were incubated with ANM (10 μM) or NAC (100 μM) in the presence of HG (30 mM) for 72 h. Cell-cycle distribution was measured by flow cytometer using PI. Percentage of cell population in each transition phase is shown in the histogram. **B.** Western blot analysis was performed to determine the expresion levels of cell-cycle regulatorty proteins including, pRb, cyclin D1, cyclin E, cyclin B1, CDK4, CDK6, CDK2 and Cdc2. GAPDH served as an internal control. **C.** To determine the effect of ANM or NAC on cell proliferation, HNDFs 5 × 10^4^ cells/well in a 6-well plates were incubated with ANM (10 μM) or NAC (100 μM) in the presence of HG (30 mM) for 72 h. Number of viable cells was quantified by the tryphan blue exclusion method. Results expressed as mean ± S.E.M of three indipendent expriments. Statisticl significance was et at ^Ф^ *P* < 0.05 compared to NG *vs* HG and *P < 0.05 compared to HG *vs.* samples.

Senescence is well-defined as an irreversible arrest in the G_0_/G_1_ phase of the cell-cycle, triggered by various physiological and chemical stimuli including HG [23]. It is thus paradoxical that HG-induced senescence is associated with cell-cycle arrest. To further explore this paradoxical relationship, we treated HNDFs with HG and ANM or *N*-acetylcysteine (NAC) for 72 h. The cell-cycle distribution pattern was determined by flow cytometry. Our results demonstrated that treatment with HG caused cell-cycle arrest in the G_1_-S transition phase, as the proportion of cells in the G_0_/G_1_ phase was significantly increased to 71.2% compared to 46.5% in the NG group. Treatment with ANM eliminated the effect of HG and reduced the cell population in the G_0_/G_1_ phase to 49.5%, which is similar to the control (NG) group (Figure 3A). However, treatment with NAC partially blocked HG-induced cell-cycle arrest in HNDFs (Figure 3A). To further clarify this effect, G_1_-S transition regulatory proteins were determined by immunoblotting. Cells exposed to HG for 72 h resulted in a significant increase in protein Rb phosphorylation and a decrease in cyclin D1, CDK4, CDK6, cyclin E and CDK2 protein levels compared to NG. However, treatment with ANM significantly inhibited protein Rb phosphorylation and upregulated cyclin D1, CDK4, CDK6, cyclin E and CDK2, whereas cyclin B1 and Cdc2 levels were unaffected (Figure 3B). Mirroring the results of the flow cytometric analysis, immunoblotting also showed that treatment with NAC partially rescued HG-induced reduction in cyclins and CDKs (Figure 3B). This result supports our observation above that treatment with ANM or NAC significantly rescued HG-mediated decrease in AKT or ERK1/2 phosphorylation, which play a functional role in cell proliferation and survival. Furthermore, cell proliferation analysis confirms that treatment with ANM protects HNDFs from HG-induced growth arrest, as indicated by increased cell proliferation (Figure 3C).

### Antcin M inhibits high-glucose-induced senescence in HNDFs by blocking ROS generation

**Figure 4:**
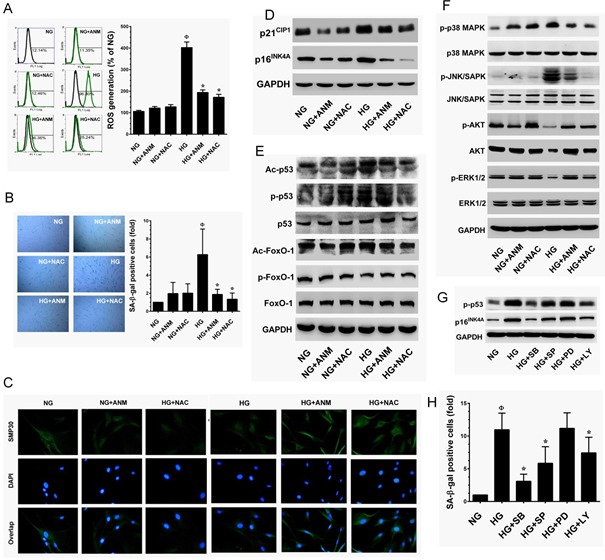
Antcin M inhibits HG-induced senescence in HNDFs **A.** ANM inhibits HG-induced intracellular ROS. HNDFs were incubated with ANM (10 μM) or NAC (100 μM) either in the presence or absence of HG (30 mM) for 24 h. The intracellular ROS level was determined by flow cytometry using DCH-DA flurogenic probe. The left panel shows representative figures and the right panel shows quantitative analysis of intracellular ROS in HG-treated HNDFs. **B.** ANM eliminates HG-induced senescenece. HNDFs were incubated with ANM (10 μM) or NAC (100 μM) in the presence or absence of HG (30 mM) for 72 h. Cellular senescence was determined by SA-β-gal assay. The left panel shows representative figures and right panel shows quantitative analysis of SA-β-gal positive cells per microscopic field. **C.** ANM provides SMP30 expression. HNDFs were incubated with ANM (10 μM) or NAC (100 μM) in the presence or absence of HG (30 mM) for 72 h. The protein expression of SMP30 was measured by immunofluroscence using SMP30 specific primary antibody and fluorescein isothiocyanate-conjugated secondary antibody (green). The cellular localization of SMP30 was photographed using a fluroscence microscope. DAPI (1 μM) was used to stain the nucleus. **D.**-**G.** To determine the effect of ANM on senescence-associated protein expression, HNDFs were incubated with ANM (10 μM) or NAC (100 μM) in the presence or absence of HG (30 mM) for 72 h. Immunoblotting analysis was used to determine the protein levels with corresponding specific antibodies. **H.** p38 MAPK, JNK/SAPK and AKT triggers HG-induced senescence. HNDFs were exposed to HG (30 mM) in the presence or absence of p38 MAPK, JNK/SAPK, ERK1/2 and AKT inhibitors SB203580 (SB, 30 μM), SP600125 (SP, 30 μM), PD98059 (PD, 30 μM) and LY294002 (LY, 30 μM) for 72 h. Cellular senescence was determined by SA-β-gal activity assay. Results expressed as mean ± S.E.M of three indipendent expriments. Statistical significance at ^Ф^*, P* < 0.05 compared to NG *vs.* HG and *P < 0.05 compared to HG *vs.* samples.

Next, we examined whether ANM inhibits HG-induced ROS generation. HNDFs were co-incubated with HG and ANM or NAC for 24 h, and intracellular ROS levels were measured by flow cytometry. Treatment with ANM or NAC alone did not significantly increase ROS generation, whereas HG-induced ROS generation (426.2%) was significantly prevented by ANM (200.8%) or NAC (176.58%) (Figure 4A). Therefore, in order to examine whether the ROS inhibitory effect may be extended to suppress HG-induced senescence, cells were co-incubated with HG and ANM or NAC for 72 h. Treatment with ANM significantly blocked the HG-induced senescence in HNDFs as evidenced by decreased number of SA-β-gal positive cells from 5.72-fold to 1.89-fold. A similar result was also observed in a pharmacological inhibitor of NAC (Figure 4B). Moreover, SMP30 has been considered to be an important protein marker of aging. Immunofluorescence analysis showed that HG-induced reduction in SMP30 was significantly prevented by ANM, compared with cells that were exposed to HG alone (Figure 4C). In contrast, NAC partially prevented the HG-induced SMP30 depletion in HNDFs (Figure 4C). In order to assess the cellular and molecular basis of the ANM-mediated inhibition of senescence, we examined the expression levels of senescence-associated marker proteins including, p16^INK4A^ and p21^Cip1^. Immunoblot analyses indicated that p16^INK4A^ and p21^Cip1^protein levels were significantly increased in the HG treatment group compared to the NG, while co-incubation with ANM significantly attenuated the expression levels of p16^INK4A^ and p21^Cip1^ proteins (Figure 4D). Indeed, treatment with ANM alone significantly reduced the basal level of p21^CIP1^ expression in HNDFs. It is well known that p16^INK4A^ and p21^Cip1^ are regulated by transcription factors p53 and FoxO1 followed by acetylation. Our result shows that treatment with HG markedly increased p53 and FoxO1 acetylation, whereas in the presence of ANM, acetylation in p53 and FoxO1 were barely observed (Figure 4E). In addition, p53 phosphorylation at Ser15 by their upstream kinases promotes transcriptional activation in response to DNA damage. Here we found that HG treatment resulted in a remarkable increase in p53 phosphorylation at Ser15, which was significantly blocked by ANM or NAC. Furthermore, the phosphorylation levels FoxO1 (p-FoxO1) significantly declined in the HG treatment group, whereas co-treatment with ANM or NAC failed to protect against the decrease in FoxO1 phosphorylation (Figure 4E). In addition, neither ANM nor NAC affected the total p53 and FoxO1 levels. Next, we examined the possible upstream regulators of p53 activation. Previous studies have shown that p38 MAPK mediated p53 activation in response to intracellular ROS generation [24]. This effect was further extended to its upstream regulator p38 MAPK, treatment with ANM or NAC significantly prevented the HG-induced activation of p38 MAPK in HNDFs (Figure 4F). HG treatment also significantly increased JNK/SAPK phosphorylation; however, co-incubation with ANM or NAC significantly prevented JNK/SAPK activation in HNDFs (Figure 4F). In addition, HG treatment caused a remarkable decrease in AKT and ER1/2 activity, whereas ANM and NAC treatment significantly blocked this effect (Figure 4F). To further examine the phenomenon that HG-induced p53 activation is relayed by the p38 MAPK or JNK/SAPK cascade, we incubated cells with corresponding pharmacological inhibitors, SB203580, SP600125, PD98059 and LY294002 for p38MAPK, JNK/SAPK, ERK1/2 and PI3K/AKT, respectively in the presence of HG. Our data showed reduced p16^INK4A^ expression and p53 phosphorylation in p38 MAPK inhibitor-treated cells, and a partial reduction in p16^INK4A^ and p53 activity was found in JNK/SAPK and PI3K/AKT inhibitor-treated cells, whereas treatment with ERK1/2 inhibitor failed to protect HG-induced p16^INK4A^ and p53 activity in HNDFs (Figure 4G). This effect was further confirmed with SA-β-gal activity assay that showed that HG-induced SA-β-gal activity was barely observed in p38 MAPK and JNK/SAPK inhibitor-treated cells, whereas inhibition of ERK1/2 did not affect the HG-induced SA-β-gal activity (Figure 4H). In contrast, inhibition of AKT also reduced the HG-induced SA-β-gal activity. These results suggest that the p38 MAPK, JNK/SAPK and PI3K/AKT cascades play a functional role in HG-induced p16^INK4A^ and p53 activation and senescence, and also suggest that ANM-mediated inhibition of p16^INK4A^ and p53 activity may be associated with suppression of p38 MAPK and JNK/SAPK activation.

### Antcin M activates Nrf2-dependent antioxidant genes in HNDFs

**Figure 5:**
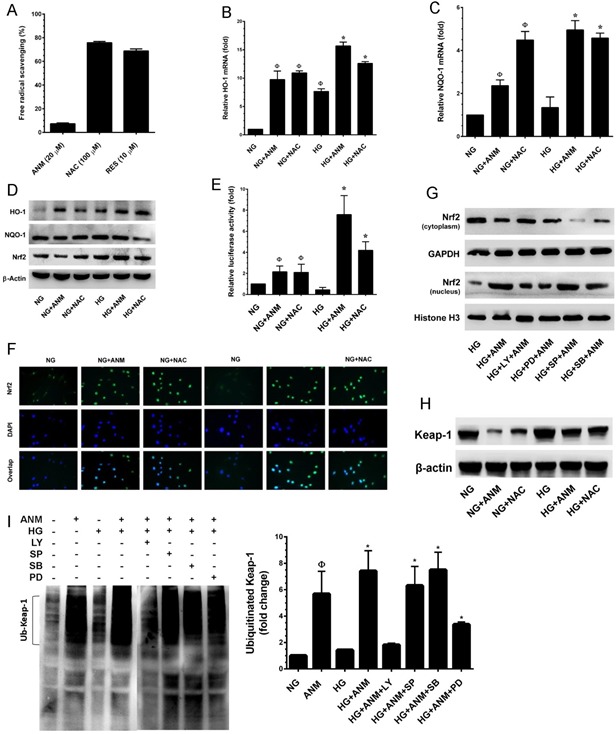
Antcin M activates Nrf2-dependent anti-oxidant defense in HNDFs **A.** To determine the free-radical scavenging effect of ANM, cell-free DPPH assay was performed. NAC and RES were used as positive controls. **B.**, **C.** To quantify the mRNA expression levels of HO-1 and NQO-1, HNDFs were incubated with ANM (10 μM) or NAC (100 μM) in the presence or absence of HG (30 mM) for 12 h. Total RNA was extracted and subjected to Q-PCR analysis. Relative mRNA levels were normalized with β-actin mRNA. **D.** To determine the protein expression levels of HO-1, NQO-1 and Nrf2, HNDFs were incubated with ANM (10 μM) or NAC (100 μM) for 24 h. Total cell lysates were prepared and subjected to western blot analysis to monitor the expression levels of HO-1, NQO-1 and Nrf2. **E.** To determine the Nrf2 transcriptional activity, HNDFs were transiently transfected with ARE promoter construct using lipofectamine and incubated with ANM (10 μM) or NAC (100 μM) in the presence or absence of HG (30 mM) for 6 h. Cell lysates were mixed with luciferase reagents and quantified using an illuminometer. Relative ARE promoter activity was calculated by dividing the relative luciferace unit (RLU) of treated cells by RLU of untreated cells (NG). **F.** To determine the nuclear localization of Nrf2, HNDFs were incubated with ANM (10 μM) or NAC (100 μM) in the presence or absence or HG (30 mM) for 2 h. The protein expression and localization of Nrf2 was measured by immunofluorescence using Nrf2 specific primary antibody and fluorescein isothiocyanate-conjugated secondary antibody (green). The subcellular and nuclear localization of Nrf2 was photographed using a fluoroscence microscope. DAPI (1 μM) was used to stain the nucleus. **G.** HNDFs were pre-incubated with AKT, ERK1/2, JNK/SAPK and p38 MAPK inhbitors LY294002 (LY, 30 μM), PD98059 (PD, 30 μM), SP600125 (SP, 30 μM) and SB203580 (SB, 30 μM), respectively for 2 h and then incubated with ANM (10 μM) in the presence of HG (30 mM) 2 h. Cytoplasmic and nuclear fractions were prepared and subjected to western blot analysis. GAPDH and histone H3 served as internal controls for the cytoplasmic and nuclear fraction, respectively. **H.** The Keap-1 protein expression level was determined by western blotting. **I.** Effect of ANM on ubiquitination of Keap-1. Equivalent amount of proteins were immune-precipitated with Keap-1 antibody and visualized by western blotting with ubiquitin antibody. Histogram shows the percentage of ubiquinated Keap-1. Results expressed as mean ± S.E.M of three indipendent expriments. Statistical significance was set at ^Ф^*P* < 0.05 compared to NG *vs.* HG or ANM alone or NAC alone and *P < 0.05 compared to HG *vs.* samples.

Above, we established that ANM inhibits ROS generation in HNDFs; however, the mechanism behind this activity was still unclear. Therefore, next to determine whether ANM acts directly as a free-radical scavenger, we performed a cell-free DPPH free-radical scavenging assay. As shown in Figure 5A, ANM failed to scavenge free radicals in the cell-free system, whereas NAC or resveratrol (RES) exhibited a potent free-radical scavenging effect. In addition, we have previously reported that antcin C (ANC), an analog of ANM induced Nrf2-dependent anti-oxidant genes in hepatic cells [19]. Therefore, we hypothesized that ANM may upregulate anti-oxidant genes, which may suppress HG-induced ROS generation in HNDFs. As we expected, treatment with ANM significantly increased the mRNA levels of phase II enzymes such as HO-1 and NQO-1 in HNDFs (Figure 5B& 5C). In contrast, compared with the NG treatment group, increased expression levels of HO-1 and NQO-1 were observed in the HG treatment group. However, treatment with ANM further increased HO-1 and NQO-1 in the HG treated groups (Figure 5B&5C). This result was further confirmed by western blotting which demonstrated that compared to the control (NG), ANM and NAC significantly increased HO-1 expression in both the NG and HG groups, whereas NQO-1 was unaffected by both ANM and NAC (Figure 5D). It is well demonstrated that anti-oxidant genes including HO-1 and NQO-1 are regulated by the transcription factor Nrf2. Therefore, to determine whether ANM augments Nrf2 transcriptional activity, we used ARE-harboring luciferase reporter assay. As shown in Figure 5E, the luciferase activity in HNDFs transfected with the ARE reporter construct was significantly increased to 5.8-fold, 6.3-fold and 2.5-fold by ANM, NAC and HG, respectively when compared to the control (1-fold). However, a remarkable increase in luciferase activity was observed in cells that were co-treated with HG and ANM or NAC which showed 8.5-fold and 8.2-fold increase, respectively. Transcriptional activation of Nrf2 is dependent upon the rate of nuclear export followed by disassociation from cytoplasmic Keap-1. Results from immunofluorescence analyses showed that Nrf2 expression in the nucleus was barely observed in the control (NG) and the HG treatment groups, whereas elevated Nrf2 expression in the nucleus was observed in the ANM or NAC treatment groups (Figure 5F). Activation of PI3K/AKT and mitogen-activated protein kinases (MAPKs) including, ERK1/2, JNK/SAPK and p38 MAPK facilitate Nrf2 transcriptional activation in a variety of human cell lines [13]. Above, we indicated that ANM significantly increased AKT and ER1/2 activities, and decreased p38 MAPK and JNK/SAPK activities (Figure 4F). To elucidate the upstream signaling events involved in ANM-induced Nrf2 transcriptional activity, cells were pre-incubated with pharmacological inhibitors of PI3K/AKT (LY294002), ERK1/2 (PD98059), SAPK/JNK (SP600125) and p38MAPK (SB203580) for 2 h and treated with ANM for 6 h in the presence of HG. In the ARE-dependent luciferase reporter system, pretreatment of cells with LY294002 and PD98059 effectively suppressed ANM-induced ARE luciferase activity, whereas pre-incubation of cells with SP600125 and SB203580 partially or barely inhibited luciferase activity (Figure 5G). These results suggest that ANM-induced Nrf2 transcriptional activity was regulated by the activation of AKT or ERK1/2 in HNDFs.

Under normal physiological condition, Nrf2 is sequestered in the cytoplasm, where it associated with Keap-1, an actin-binding protein. Upon chemical treatment or oxidative stress conditions, the steady-state levels of Keap-1 is rapidly degraded through the ubiquitin-dependent proteasome pathway, which eventually causes Nrf2 accumulation and transcriptional activity. To determine whether the up-regulated ratio of Nrf2 in nucleus by ANM is due to the induction of Keap-1 ubiquitination, we examined the ubiquitination of Keap-1 by immunoprecipitation after treatment with ANM in the presence or absence of HG. As shown in Figure 5H, the Keap-1 protein level was significantly decreased after treatment with ANM alone or in HG-induced condition. On other hand, a significant increase in ubiquitination of total protein was observed in cells treatment with ANM or NAC (data not shown). After immunoprecipitation with anti-Keap-1 antibody, a remarkable increase of ubiquitination of Keap-1 was observed in cells treatment with ANM (Figure 5I), and further increase was observed when cells were co-incubated with HG (Figure 5I). This data suggest that up-regulation of Nrf2 protein by ANM was due to the enhancement of Keap-1 ubiquitination, and has the possibility that ANM may directly or indirectly induce Keap-1 ubiquitination. Our data also indicated that up-regulation of Nrf2 was mediated by AKT and ERK1/2 (Figure 5G). Therefore, we further examined whether AKT and ERK1/2 have any influence on Keap-1 ubiquitination, cells were co-incubated with AKT, p38MAPK, JNK and ERK1/2 inhibitors in the presence of ATM and the Keap-1 ubiquitination was examined. As shown in Figure 5I, ANM-induced Keap-1 ubiquitination was markedly observed in p38MAPK or JNK1/2 inhibitors treated cells, whereas a reduced levels of Keap-1 ubiquitination was noted in AKT and ERK inhibitor treated cells. These data confirm that ANM-induced activation of AKT or ERK1/2 induce Keap-1 proteasome degradation in HNDFs.

### Antcin M failed to protect HG-induced oxidative stress in Nrf2 silenced cells

**Figure 6:**
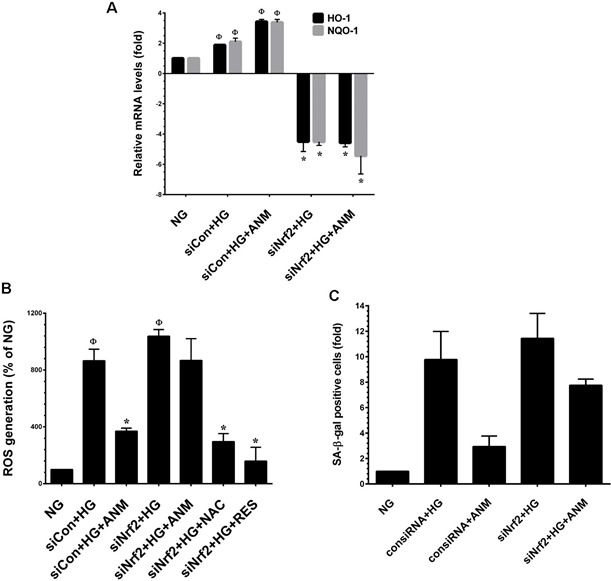
Antcin M failed to protect HG-induced oxidative stress in Nrf2 silenced cells **A.** HNDFs were transfected with specific siRNA against Nrf2 or control siRNA. After transfection for 24 h, cells were incubated with ANM (10 μM) in the presence of HG for 12 h. Total RNA was extracted and subjected to Q-PCR analysis to determine HO-1 and NQO-1 mRNA expression levels. **B.** HNDFs were transfected with specific siRNA against Nrf2 or control siRNA. After transfection for 24 h, cells were incubated with ANM (10 μM) or NAC (100 μM) or RES (5 μM) in the presence of HG for 24 h. Intracellular ROS was measured by DCFH-DA assay. **C.** HNDFs were transfected with specific siRNA against Nrf2 or control siRNA. After transfection for 24 h, cells were incubated with ANM (10 μM) in the presence of HG for 72 h. SA-β-gal activity was measured. Results expressed as mean ± S.E.M of three indipendent expriments. Statistical significance was set at ^Ф^*, P* < 0.05 compared to NG *vs.* HG or ANM alone or NAC alone and *P < 0.05 compared to HG *vs.* samples.

To confirm our hypothesis that ANM protects HNDFs from HG-induced oxidative stress, we developed an Nrf2 gene knockdown system using Nrf2 siRNA. As shown in Figure 6A, a partial increase in the expression levels of HO-1 and NQO-1 mRNA were observed in scrambled siRNA (control siRNA) transfected cells, and co-incubation with ANM exhibited a remarkable increase in HO-1 and NQO-1 mRNA levels. Although treatment with HG alone or along with ANM showed a decrease in HO-1 and NQO-1 expression in siNrf2-transfected cells, indeed, the HO-1 and NQO-1 mRNA levels declined below the basal level in siNrf2 transfected cells. From this data, it can be concluded that Nrf2 plays a vital role in HO-1 and NQO-1 induction even at the basal level. Moreover, treatment with ANM significantly inhibited HG-induced ROS generation in scrambled siRNA transfected cells, whereas increased ROS generation was observed in siNrf2 transfected cells even after treatment with ANM (Figure 6B). To further clarify this protective effect, HG-induced senescence was measured by SA-β-gal assay. In control siRNA transfected cells, treatment with ANM significantly inhibited HG-induced senescence. In contrast, treatment with ANM significantly prevented HG-induced senescence in siNrf2-transfected cells (Figure 6C). This data suggests that ANM-induced activation of the Nrf2-dependent antioxidant mechanism at least partially supports the protective effect of ANM; however, there may be other possible mechanisms involved in the complete protection provided by ANM.

### Antcin M upregulates SIRT-1 in HNDFs

**Figure 7:**
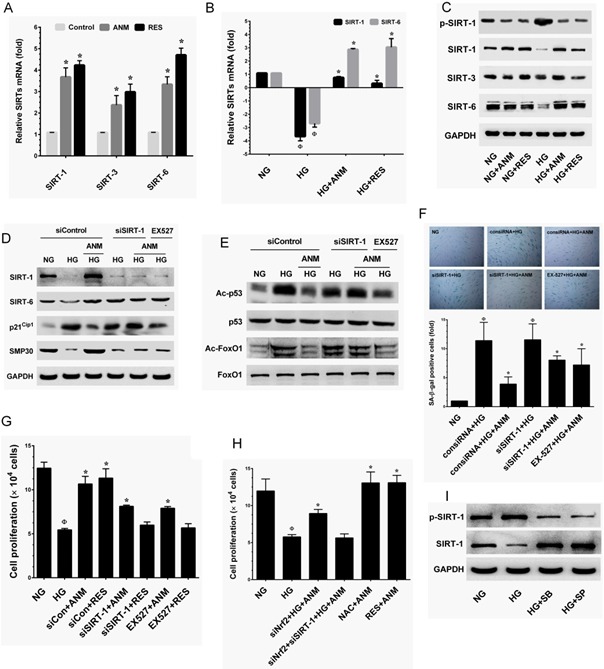
Antcin M upregulates SIRT-1 in HNDFs **A.** HNDFs were incubated with ANM (10 μM) or RES (5 μM) for 72 h. **B.** HNDFs were exposed to HG in the prsence or absence of ANM (10 μM) or RES (5 μM) for 72 h. Total RNA was extracted and subjected to Q-PCR analysis to monitor SIRT-1, SIRT-3 and SIRT-6 expression. Relative mRNA levels were normalized by β-actin mRNA. **C.** HNDFs were incubated with ANM (10 μM) or RES (5 μM) in the presence or absence of HG (30 mM) for 72 h. Total cell lysate was extracted and subjected to western blot analysis to monitor SIRT-1, SIRT-3, SIRT-6 protein levels. **D.**, **E.** HNDFs were transfected with siRNA against SIRT-1 or control siRNA for 24 h or inhibited by SIRT-1 inhibitor EX527 (5 μM), and then treated with ANM (10 μM) or RES (5 μM) in the presence of HG for 72 h. The protein expression levels of SIRT-1, SIRT-6, p21CIP1, SMP30, p53, FoxO and acetylation of p53 and FoxO1 were determined by western blot analysis. **F.**, **G.** Under the same conditions, cellular senescence and cell proliferation were measured by SA-β-gal activity assay and tryphan blue exclusion assay, respectively. **H.** HNDFs were transfected with siNrf2 or a combination of siNrf2 and siSIRT-1, and then incubated with ANM in the presence or absence of HG for 72 h. Cell proliferation was determined by tryphan blue exclussion assay. **I.** Cells were incubated with JNK/SAPK or p38 MAPK inhibitors SP600125 (SP, 30 μM) and SB203580 (SB, 30 μM) in the presence of HG for 72 h. The protein expression levels of phos-SIRT-1 and SIRT-1 were determined by western blotting. Results expressed as mean ± S.E.M of three indipendent expriments. Statistical significance at ^Ф^*, P* < 0.05 compared to NG *vs* HG and *P < 0.05 compared to HG *vs.* samples.

To determine whether ANM regulates HNDF senescence through a SIRT-1-mediated pathway, we examined the expression levels of SIRT genes SIRT-1, SIRT-3 and SIRT-6. As shown in Figure 7A, RT-PCR analysis indicated that SIRT-1, SIRT-3 and SIRT-6 levels were significantly increased in the ANM treatment group compared to the control group. SIRT-1 and SIRT-3 expression levels were highly comparable to the known SIRT-1 activator resveratrol (RES). In addition, treatment with ANM also significantly increased SIRT-6, whereas a remarkable increase was observed in the RES treatment group (Figure 7A). Previous studies have shown that exposure of endothelial cells to HG rapidly decreased levels of expression of SIRT genes [25, 26]. Our results also demonstrate that exposure of HNDFs to HG markedly decreased SIRT-1 and SIRT-6 expression compared to that of cells exposed to NG, whereas treatment with ANM rescued SIRT-1 and SIRT-6 from HG-induced depletion (Figure 7B). Immunoblotting further confirmed that ANM significantly prevented HG-induced reduction in SIRT-1, SIRT-3 and SIRT-6 proteins (Figure 7C).

Since SIRT-1, a NAD^+^-dependent class III histone deacetylase has been shown to interact with a number of molecules including p53 and FoxO1 [14, 15]. As shown in Figure 4E, treatment with ANM significantly modulated the HG-induced acetylation in p53 and FoxO1. Next, to investigate whether the deacetylation activity of ANM is SIRT-1-dependent, deacetylation activity of ANM was determined under SIRT-1 silenced conditions. In control siRNA (scrambled siRNA) transfected cells, ANM significantly increased SIRT-1 and SIRT-6 expression, whereas SIRT-1 not SIRT-6 was barely observed in SIRT-1 silenced cells (Figure 7D). Moreover, in control siRNA transfected cells, the HG-induced expression of p21^CIP1^ (Figure 7D), acetylation in p53 and FoxO1 (Figure 7E) were significantly attenuated upon treatment of ANM with increased SMP30 expression (Figure 7D), compared with HG alone. However, treatment with ANM failed to inhibit the p21^CIP1^ expression and deacetylation in p53 and FoxO1 or upregulation of SMP30 in SIRT-1 silenced cells (Figure 7D&E). Furthermore, a similar effect was also observed in SIRT-1 inhibitor (EX527)-treated cells (Figure 7D&7E).

In order to ascertain whether the protective effect of ANM was SIRT-1 dependent, the effect of ANM in SIRT-1 silenced HNDFs was investigated under HG conditions. In control siRNA transfected cells, treatment with ANM significantly inhibited HG-induced senescence as assessed by SA-β-gal activity. However, in SIRT-1 siRNA transfected cells, SA-β-gal activity remained partially elevated despite the presence of ANM or RES (Figure 7F). Indeed, compared with the HG alone treatment group, ANM showed a significant inhibition of SA-β-gal activity although in the SIRT-1 silenced cells (Figure 7F). Likewise, cell proliferation analysis also indicated that, in control siRNA transfected cells, the HG-induced reduction in cell number was significantly blocked by ANM, whereas partial protection was observed in SIRT-1 siRNA transfected cells (Figure 7G). In addition, a similar effect was also observed in SIRT-1 inhibitor (EX527)-treated cells (Figure 7G). These data strongly suggest that SIRT-1 partially contributes to the protective effects of ANM. Interestingly, HG-induced reduction in cell proliferation was partially inhibited by ANM in Nrf2 knock-down cells, whereas ANM failed to rescue cell proliferation in Nrf2 and SIRT-1 knock-down cells (Figure 7H). Furthermore, complete protection was achieved by co-treatment with ANM and NAC or RES (Figure 7H). These data strongly suggest that ANM-mediated anti-oxidant defense and SIRT-1-mediated deacetylation activity regulates HG-induced senescence in HNDFs.

### Antcin M prevents HG-induced SIRT-1 degradation via suppression of p38 MAPK and JNK1/2 activation

To further understand the regulation of SIRT-1 by ANM, we examined the effect of ANM on SIRT-1 activation and protein stability under hyperglycemic conditions. Previous studies have shown that hyperphosphorylation of SIRT-1 at serine 47 (Ser47) was correlated with enhanced endothelial senescence [16]. In addition, persistent activation of JNK1/2 by multiple factors including hyperglycemia induces extensive SIRT-1 proteasome degradation followed by phosphorylation at Ser47 [27]. In the present study we found that a remarkable increase in SIRT-1 phosphorylation at Ser47 was observed after exposure to HG. However, treatment with ANM or RES significantly attenuated this effect (Figure 7I). As shown in Figure 4F, JNK1/2 and p38 MAPK activity was increased by HG as indicated by increase in their phosphorylation, whereas ANM treatment significantly prevented HG-mediated JNK1/2 and p38 MAPK activation in HNDFs. Therefore, we hypothesize that ANM-mediated suppression of JNK1/2 and p38 MAPK activation may have a functional role in the stability of SIRT-1 protein. Interestingly, Suppression of JNK1/2 and p38 MAPK activity by a pharmacological inhibitor of JNK1/2 SP600125 and p38 MAPK SB203580 inhibits SIRT-1 phosphorylation and reduction in SIRT-1. These data suggest that SIRT-1 reduction is related to JNK1/2 activation.

### Anticin M protects *Caenorhabditis elegans* from oxidative stress

**Figure 8:**
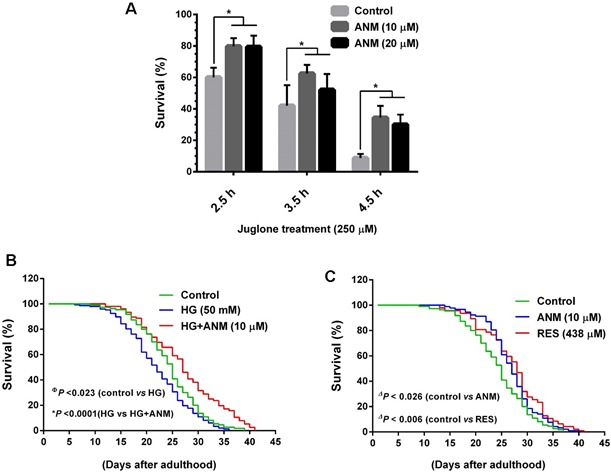
Antcin M protects wild-type *C. elegans* from oxidative stress and extent life span **A.** To determine oxidative stress resistence, age synchronized wild-type L1 larvae were pretreated with ANM (10 and 20 μM) or DMSO (0.1%) for 3 days. Oxidative stress was induced by incubation of pre-treated worms with 250 μM Juglone for 2.5, 3.5 and 4.5 h and then scored for viability. Results expressed as mean ± S.E.M of triplicate assays. Statistical significance at ^*^P < 0.05 compared to control *vs.* sample treatment. **B.** Effect of ANM on the life span of *C. elegans* under hyperglycemic condition. Age synchronized L1 larvae were cultured in NGM plates wich contain HG (50 mM) with or with out ANM (10 μM) and worm were developed to adulthood. The survival rate was scored everyday and is expressed as a percentage of survival. **C.** Effect of ANM (10 μM) or RES (438 μM) on the life span extention of *C. elegans* under normal condition. Results expressed as mean ± S.E.M of three indipendent expriments. Statistical significance at ^Ф^*P* < 0.05 compared to control *vs* HG, *P < 0.05 compared to HG *vs* HG+sample and ^Δ^*P* < 0.05 compared to control *vs* samples.

To further confirm the antioxidative potential of ANM *in vivo*, we subjected *C. elegans* model. Wild-type N2 worms were pretreated with ANM for 3 days followed by exposure to oxidative stress. Briefly, age synchronized L1 larvae were pre-treated with ANM (10 and 20 μM) or 0.1% DMSO (vehicle control) for 3 days before being exposed to Juglone (250 μM), and then incubated for 2.5, 3.5 and 4.5 h. After incubation the survived worms were scored. The result showed that pretreatment with 10 μM ANM significantly increased the survival rate of worms exposed to oxidative stress induced by Juglone (Figure 8A), demonstrating that ANM protects *C. elegans* from oxidative stress injury *in vivo*. It was noted that above 10 μM ANM pretreatment showed a similar effect on oxidative stress resistance to the worms (Figure 8A).

### Antcin M extends the life span of wild-type *C. elegans* under hyperglycemic condition

It has been well documented that high glucose levels decrease the life span of *C. elegans* by increasing ROS formation and advanced glycation end-product modification of mitochondrial proteins. Therefore, we further investigated whether ANM has a protective effect against hyperglycemia-induced oxidative stress as well as anti-aging effects, worms were incubated with high glucose (50 mM) with or without ANM (100 μM), and controlled for life span evaluation. As shown in Figure 8B, treatment with high glucose markedly decreased life span of *C. elegans*, whereas a significant (*P* < 0.0001) increase of life span was observed in co-treatment with ANM. In addition, we observed that ANM alone treatment significantly (*P* < 0.026) prolonged the life span of *C. elegans* compared to the control (Figure 8C), suggesting that ANM has a protective effect against hyperglycemia-induced oxidative stress and decreased life span. The effects of ANM were highly comparable with the well-known anti-aging reagent resveratrol (Figure 8C); both compounds were originated from natural resources.

### Antcin M prevents hyperglycemia-induced endothelial cells senescence through Nrf2/SIRT-1 activation

**Figure 9:**
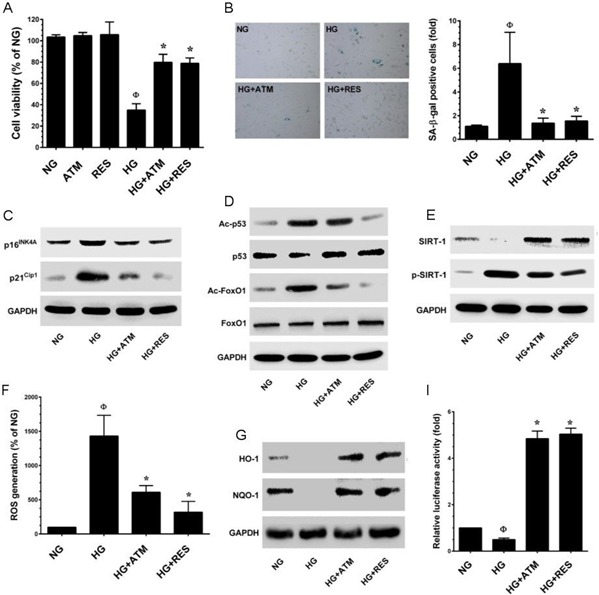
Antcin M prevents HG-induced senescence in HUVECs **A.** HUVECs were incubated with ANM (10 μM) or RES (5 μM) in the presence or absence of HG (30 mM) for 48 h. Cell viability was dermined by MTT assay. Percentage of viable cells were normalized with control cells (NG). **B.** Cellular senescence was determined by SA-β-gal activity assay as descrided in Materials and Methods. **C.**, **D.** Total cell lysate was extracted and senescence-associated marker proteins including p16INK4A, p21CIP1, total and acetylated p53 and FoxO1 were measured by western blot analysis. **E.** Protein expression levels of SIRT-1 and phos-SIRT-1 were monitored by immunoblotting. **F.** HUVECs were incubated with ANM (10 μM) or RES (5 μM) in the presence or absence of HG (30 mM) for 48 h. The intracellular ROS level was quantified by utilizing DCFH-DA assay. **G.** Western blot analysis was performed to determine the protein expression levels of HO-1 and NQO-1. **H.** HUVECs were transiently transfected with ARE promoter construct using lipofectamine and incubated with ANM (10 μM) or RES (5 μM) in the presence or absence of HG (30 mM) for 6 h. Cell lysates were mixed with luciferase reagents and quantified using an illuminometer. Relative ARE promoter activity was calculated by dividing the relative luciferace unit (RLU) of treated cells by RLU of untraeated cells (NG). Results expressed as mean ± SEM of three indipendent expriments. Statistical significance at ^Ф^*P* < 0.05 compared to NG *vs.* HG and *P < 0.05 compared to HG *vs.* samples.

To further delineate the protective effects of ANM on another cell system, experiments were designed to investigate the protective effect of ANM on human umbilical vein endothelial cells (HUVECs) incubated in media containing either NG or HG alone or with ANM for 48 h. Cell viability was measured by MTT assay. As shown in Figure 9A, treatment of HUVECs with ANM (10 μM) or RES (5 μM) for 48 h did not affect cell viability. However, exposure of HUVECs to HG (30 mM) for 48 h reduced number of viable cells to 35.01%, whereas co-incubation with ANM or RES significantly increased the number of viable cells to 79.78% and 78.79%, respectively. In addition, SA-β-gal staining was significantly increased (6.38-fold) in HG-treated HUVECs, compared with HUVECs maintained in NG, whereas treatment with ANM showed reduced endothelial senescence (1.36-fold), compared with untreated HUVECs maintained in HG (Figure 9B). In addition, the result from the western blot analysis also revealed that exposure of HUVECs to HG caused increased expression of p16^INK4A^ and p21^CIP1^ proteins and p53 and FoxO1 acetylation, compared with HUVECs maintained in NG, whereas treatment with ANM significantly blocked the HG-induced p53 and FoxO1 acetylation in HUVECs (Figure 9C, 9D). To determine whether ANM regulates HUVEC senescence through a SIRT-1-mediated pathway, we examined the protein expression levels of SIRT-1. Concomitant with HNDFs, SIRT-1 levels were significantly decreased in the HG treatment group compared to the NG, and ANM treatment significantly rescued SIRT-1 expression in HUVECs. We also found that HG treatment markedly increased SIRT-1 phosphorylation at Ser47, whereas co-treatment with ANM significantly blocked HG-induced SIRT-1 phosphorylation. A similar effect was also observed in RES-treated cells (Figure 9E). To further examine whether HG induces ROS generation which triggers endothelial senescence, ROS was measured by DCF fluorescence assay. The production of intracellular ROS was significantly increased in HUVECs after exposure to HG (14.3-fold). However, treatment of HUVECs with ANM resulted reduced ROS levels (6.1-fold), compared with HUVECs in HG that were not treated with ANM (Figure 9C).

## DISCUSSION

Cellular senescence is an inevitable process by which cells irreversibly exit the cell-cycle and stop dividing in response to a variety of stresses including those observed during hyperglycemic states. In the present study, we modeled human premature skin aging *in vitro*by culturing normal human dermal fibroblasts (HNDFs) with high-glucose (30 mM) to investigate the protective role of antcin M in cell senescence.

New therapeutic agents from natural sources have potential pharmacological properties for complicated human diseases such as diabetes and aging. Several phytochemicals including phenolic compounds, flavonoids and terpenoids exhibited anti-diabetic and anti-aging properties through their anti-oxidant or anti-inflammatory effects [28–30]. Antcins (ANA, ANB, ANC, ANH, ANK and ANM) are naturally occurring triterpenoids reported to have anti-oxidant and anti-inflammatory effects; therefore, they might have beneficial effects on diabetic mellitus and aging [18]. Initial cytotoxic assessment showed that ANA, ANC, ANH and ANM are not cytotoxic to the HNDFs at high concentrations (20 μM). However, ANB and ANK were highly toxic to the HNDFs, which is agreement with previous studies that found that ANB and ANK were cytotoxic to the hepatoma cell line [31, 32]. Therefore, next we examined the anti-aging effect of ANA, ANH and ANM. Stress-induced premature senescence (SIPS) was evident in fibroblasts incubated in hyperglycemic (>25 mM) medium, compared to those cultured in medium containing physiological concentration of glucose (5.5 mM) [10], confirming the paradoxical relationship between glucose concentration and SIPS by observation of various characteristic features of cellular senescence. Senescence-associated β-galactosidase (SA-β-gal) activity was significantly increased by hyperglycemia, suggesting that cells experienced senescence, whereas the hyperglycemia-induced increase in SA-β-gal positive cells was significantly inhibited by ANA, ANH and ANM. However, a highly pronounced inhibitory effect was observed in ANM-treated cells. Immunoblotting showed that ANA, ANH and ANM significantly down-regulated the HG-induced increase in p16^INK4A^, a tumor suppressor protein known to transduce senescence-signals and lead to irreversible growth arrest [33]. In addition, ANA, ANH and ANM significantly inhibited HG-induced p21^CIP1^ expression, a cyclin-dependent kinase inhibitor that regulates growth-arrest and cellular senescence [24]. Regucalcin, also known as SMP30 is a 34-kDa cytosolic marker protein of cellular aging, which rapidly losses its expression during senescence [22]. Our study shows a significant decrease in the levels of SMP30 in HNDFc that had been cultured in HG for 72 h, whereas in the presence of antcins, restored SMP30 expression. Taken together, our findings suggest that compared to other antcins, ANM exerts a potent beneficial effect on hyperglycemia-induced senescence through modulating the p16^INK4A^ and p21^CIP1^ pathways.

The results of the current study and previous studies [10], indicate that glucose at a concentration of 30 mM is sufficient to induce SIPS onset in dermal fibroblasts. Although, hyperglycemia enhances ROS production, which causes oxidative damage and chronic ailments including diabetes [34]. In this study, we found that HNDFs exposed to hyperglycemia exhibited characteristics associated with aging *via* increased ROS production and SA-β-gal activity. However, co-incubation with ANM robustly attenuated ROS generation and SA-β-gal activity. The relevant role of oxidative stress in senescence is demonstrated by the fact that treatment with anti-oxidants delays or eliminates cellular senescence [24]. Mechanically, excessive intracellular ROS levels leads to increased transcriptional activity of p53 through the acetylation at Lys382, which eventually up-regulates p21^CIP1^ [24]. The human endothelial cells cultured in hyperglycemic medium showed marked SA-β-gal activity in association with increased DNA damage markers, p16^INK4A^, p21^CIP1^ and p53 [25]. The present study is evidence that exposure of HNDFs to HG for 72 h, increases in the expression of p21^CIP1^ protein and increases p53 acetylation. This is the first report indicating HG-induced p53 acetylation in dermal fibroblasts. This finding is in accordance with a previous study that reported hyperglycemia accelerates p53 acetylation through intracellular ROS accumulation [25]. We, therefore, examined the hypothesis that ANM would inhibit HG-induced p53 acetylation in HNDFs. Our data demonstrated that co-treatment with ANM significantly attenuated HG-induced p53 acetylation and decreased the expression of p21^CIP1^ protein. In parallel, HNDFs cultured in HG showed a significant increase in FoxO1 acetylation. The increase in FoxO1 acetylation may result in the transcriptional activation of FoxO1 towards the transcription of cell-cycle arrest genes, which are stimulated with HG-induced oxidative stress. However, the presence of ANM significantly attenuated the FoxO1 acetylation in HNDFs exposed to HG. In addition, phosphorylation of FoxO1 at Thr24 by AKT promoted cell survival by regulating cell-cycle progression. Our data also show that HG treatment caused a remarkable decrease in FoxO1 phosphorylation, and in the presence of ANM, HG failed to abrogate FoxO1 phosphorylation in HNDFs. These data support the hypothesis that ANM provokes HG-induced senescence through the negative regulation of p53 and FoxO1. To the best of our knowledge, this is the first report indicating hyperglycemia-induced p53 and FoxO1 acetylation in dermal fibroblasts. In addition, it is well demonstrated that activation of JNK1/2 by ROS triggers p53 activation [35]. However, this pathway involved in premature senescence was poorly elucidated. In the present study, an aberrant activation of JNK1/2 was found in HG-treated cells, whereas the JNK1/2 phosphorylation was barely observed in ANM and NAC, and ROS inhibitor treated cells. These data suggest that HG-induced ROS might trigger JNK1/2 activation, which may lead to p53 activation and premature senescence.

The number of stimuli known to induce SIPS is constantly increasing and the mechanism has been extensively studied [1, 6, 7]. Increased senescence has been shown to be associated with the expression of p16^INK4A^ protein in endothelial cells cultured in hyperglycemic medium, this effect was blocked by stachydrin, a proline betaine found in citrus juice [36]. Our data shows that HG-induced increase of p16^INK4A^ significantly blocked by ANM. Several reports have shown that the ability of ROS to induce p16^INK4^ depends on p53 activation *via* its upstream kinase p38 MAPK. Constitutive activation of this pathway induces p16^INK4A^ and p21^CIP1^ and leads to premature senescence [24, 37]. Robust activation of p38 MAPK was observed in HG-treated cells, and this activation was significantly blocked by ANM. This result suggests that ANM exerts a beneficial effect on hyperglycemia-induced senescence through modulating the p16^INK4A^ and p38 MAPK cascades. Likewise, intracellular ROS activates JNK/SAPK, which triggers p53 transcriptional activity [35]. However, the link between JNK/SAPK and p16^INK4A^ remains unknown. In the present study, we found that inhibition of JNK/SAPK activity by pharmacological inhibitor resulted in reduced p16^INK4A^ protein and p53 activation, when compared to cells that were treated only in HG. To the best of our knowledge, this is the first data demonstrating the link between JNK/SAPK and p16^INK4A^.

Cell-cycle arrest and senescence is a frequently discussed topic in aging-related research. Blagosklonny [38] extensively reviewed the difference between quiescence and senescence. Quiescent cells are capable of restarting proliferation by addition of growth factors. Nevertheless, senescent cells arrested at G_0_/G_1_ phase and inability to restart proliferation. In line with the previous studies [36], HG arrested cells in G_1_-S transition phase, and increased cell population in the G_0_/G_1_ phase. This effect was blocked by co-treatment with ANM which kept the percentage of cells in the G_0_/G_1_ phase near to control values. Cell-cycle progression is regulated by complexes of cyclins and cyclin-dependent kinases (CDKs), and reduction in the complex of cyclin D with CDK4/CDK6 and cyclin E with CDK2 resulted in G1-S transition arrest [39]. In addition, disruption of cyclin/CDK complex promotes retinoblastoma protein (pRb) stability and prevents the progression from the G_1_ to S phase of the cell division *via* inhibiting the transcription factor E2F family which plays a major role in G_1_-S transition in mammalian cells [39]. In the present study, we found that treatment with HG resulted in decreased pRb phosphorylation followed by reduction in cyclin D1, CDK4, CDK4, cyclin E and CDK2, which was eliminated following co-treatment with ANM. The results obtained from cell-cycle analysis were consistent with this observation.

Eukaryotic cells are fortified with primary and secondary defense against oxidative stress insults. Particularly, the phase II enzymes such as hemeoxygenase-1 (HO-1), NAD(P)H: quinone oxidoreductase 1 (NQO1), and glutathione-*S*-transferase (GST) are rapidly activated by an endogenous mechanism through which oxidative toxicants can be removed before they damage DNA [13]. Many natural products have been reported to have beneficial effects on the aging processes: polyphenols, flavonoids, terpenoids, caratinoids, vitamins, resveratrol, curcumin, ferulic acid and coffeic acid, are well-known for their high anti-oxidant content. These components act not only as free radical scavengers but also by modulating signal transduction pathways and gene expression patterns [12]. In this study, ANM showed strong inhibition of HG-induced ROS generation, which demonstrated the anti-oxidant efficacy of ANM. Our further analysis revealed that ANM does not have a direct free-radical scavenging effect as measured by DPPH assay. A previous study showed that antcin C, a similar analog of ANM exerted free-radical-induced oxidative stress in hepatocytes through the induction of Nrf2-dependent anti-oxidant genes [19]. However, ANM eliminates exsecive ROS generation through the induction of anti-oxidant genes such as HO-1 and NQO-1. The increased levels of anti-oxidant genes were observed after co-treatment with HG and ANM. This data suggests that ANM induces anti-oxidant genes upon excessive oxidative stress. In contrast, treatment with HG also increases HO-1 and NQO-1 mRNA levels, however the NQO-1 expression under HG was not statistically significant with control cells. Nrf2, a bZIP transcription factor, regulates the expression of anti-oxidant genes including HO-1 and NQO-1 [40]. Under normal physiological conditions, Nrf2 is sequestrated in the cytoplasm, and upon stimulation, disassociates from its cytosolic inhibitor Keap-1, translocates into the nucleus and binds to the *cis*-acting anti-oxidant responsible element (ARE) in the promoter region [13, 40]. Many studies have shown that ARE promoter was targeted by dietary phytochemicals as evidenced by the finding that deletion of ARE-site containing E1 and E2 regions blunts induction [13, 40]. In this study, we demonstrated that treatment with ANM significantly increased the transcriptional activity of Nrf2 in HG-induced HNDFs.

Senescence-related hyperglycemia is associated with increased oxidative stress *via* MAPKs [10]. Moreover, the transcription factor Nrf2 is activated by upstream kinases including PI3K/AKT, PKC, JNK/SAPK, ERK1/2 and p38 MAPK [13]. In this study, PI3K/AKT and ERK1/2 were significantly up-regulated by ANM under normal and hyperglycemic conditions, which may be associated with Nrf2 activation. Result showed that ANM-induced Nrf2 transcriptional activity was significantly abolished by PI3K/AKT and ERK1/2 inhibitors, which demonstrates that ANM-induced Nrf2 activity was mediated by the PI3K/AKT and ERK1/2 cascades. Indeed, a remarkable increase in Nrf2 activity was observed in JNK/SAPK inhibitor treated cells supporting the notion that JNK/SAPK downregulates Nrf2 activity in HG-treated cells. This data is consistent with our previous study that antcin C induces Nrf2 activity *via* activation of the PI3K/AKT and JNK/SAPK pathways [19]. Moreover, the activation of Nrf2 by phytochemicals is involved in various upstream mechanisms. For example, curcumin, coffeic acid and suphoraphane directly target the thiol group of Keap-1 to induce proteasomal degradation, which promotes Nrf2 transcriptional activity [13]. Furthermore, silencing Nrf2 by siRNA failed to protect HG-induced cellular senescence even in the presence of ANM, demonstrating the role of Nrf2-mediated anti-oxidant mechanism in oxidative stress-induced premature senescence.

A growing body of evidence suggests that SIRT-1 is an important modulator of cellular senescence, longevity, metabolism and apoptosis. Previous studies show that inhibition of SIRT-1 by sirtinol or SIRT-1 siRNA result in a premature senescence-like phenotype in endothelial and young mesenchymal stem cells and overexpression of SIRT-1 reversed this processes [41, 42]. In addition, hyperglycemia accelerates endothelial cell senescence which is associated with reduction in SIRT-1 [25]. These studies imply that SIRT-1 plays a pivotal role in regulation of cellular senescence. In our study, we found that treatment with ANM alone could increase SIRT-1 mRNA expression along with SIRT-3 and SIRT-6. In line with a previous study [25], exposure of HNDFs to HG caused a dramatic reduction in SIRT-1 and SIRT-6 mRNA and protein expression levels. However, co-incubation with ANM counteracts the detrimental effects of HG by upregulating SIRT-1 and SIRT-6 expression levels. It has been reported that hyper-phosphorylation of SIRT-1 at Ser47 by JNK/SAPK induces proteasome degradation of SIRT-1 in fibroblasts [27]. However, other factors involved in SIRT-1 phosphorylation at Ser47 are poorly understood. Interestingly, our data shows that treatment with HG increased JNK/SAPK activation as well as SIRT-1 phosphorylation in HNDFs. This connection was further confirmed by observation of very little HG-induced SIRT-1 depletion and SIRT-1 phosphorylation in JNK/SAPK inhibitor-treated cells. A similar effect was also observed in p38 MAPK inhibitor-treated cells. These data confirm that HG-induced SIRT-1 depletion was coordinated by JNK/SAPK and p38 MAPK *via* increased SIRT-1 hyper-phosphorylation and proteasome degradation. Previous studies suggested that the protective effect of SIRT-1 may be due to the regulation of acetylation/deacetylation of key transcription factors such as p53 and FoxO1 [14, 15]. Activation of p53 by external or internal stimuli induces expression of several genes including p21^CIP1^ and p16^INK4A^, which are bound to the G_1_-S transition kinases (CDK4, CDK6, CDK2 and CDK1) and inhibit their activity [43]. Likewise, FoxO1 transcription factor plays a crucial role in cellular senescence by upregulating p21^CIP1^ and p16^INK4A^ genes [44]. In this study, HNDFs exposed to HG showed a significant decrease in SIRT-1 expression and a parallel significant increase in p53 and FoxO1 acetylation. This increase in p53 and FoxO1 acetylation may result in the switching of p53 and FoxO1 transcriptional activity towards transcription of growth inhibition or senescence inducible genes. The presence of ANM significantly reduced p53 and FoxO1 acetylation with a subsequent decrease in p21^CIP1^ and p16^INK4A^. Our data also show that treatment with ANM did not significantly attenuate the p53 and FoxO1 acetylation and p21^CIP1^ and p16^INK4A^ expression in SIRT-1-silenced HNDFs exposed to HG. Surprisingly, treatment with ANM partially protected HG-induced cellular senescence and cell survival in SIRT-1 knock-down cells, which further suggests that the protective effect of ANM is through its anti-oxidative properties. This was confirmed by the fact that ANM failed to protect HG-induced senescence and growth arrest in SIRT-1 and Nrf2 knock-down cells. Similarly, a synergistic effect was observed when ANM was combined with a well-known antioxidant NAC and SIRT-1 enhancer resveratrol.

To further understand the effects of ANM *in vivo*, herein we used *C. elegans* as an *in vivo* model to examine the protective and anti-aging effects of ANM. There are number of studies demonstrated that the protective actions of phytochemicals in *C. elegans* are mainly attributed to their antioxidative potential [45, 46]. We showed that the survival rate of wild-type worms were significantly increased with ANM treatment under Juglone-induced oxidative stress condition, suggesting that ANM has strong antioxidative activity *in vivo*. Schulz et al [47] reported that *C. elegans* raised under high glucose condition lost the ability to oxidize glucose and suffered reduced fertility and decreased total progeny production. In addition, glucose enriched diet had significantly decreased *C. elegans* life span due to increased ROS formation [48]. We also found a reduction in the life span under a high glucose condition, whereas co-treatment with ANM significantly increased life span, suggesting that ANM has a protective effect against HG-induced oxidative stress.

**Figure 10:**
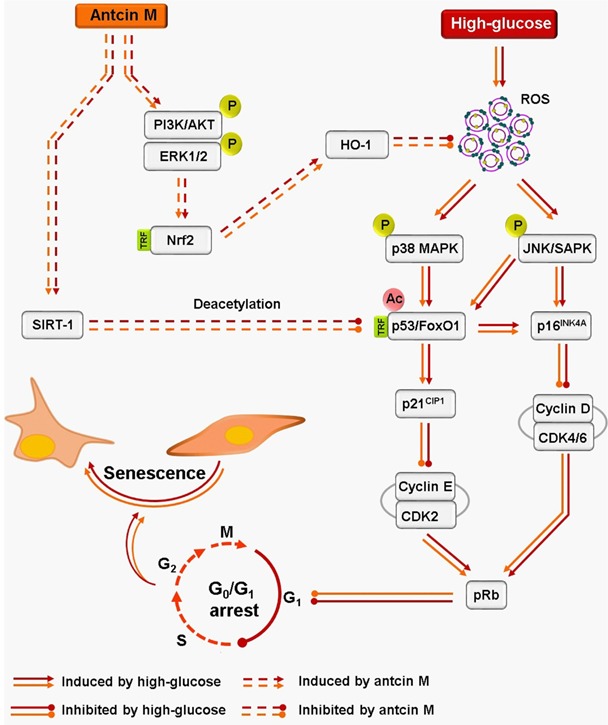
Schematic representation of antcin M-mediated protection against HG-accelarated stress-induced premature senescence in HNDFs and HUVECs Hyperglycemia induces intracellular ROS, which triggers p38 MAPK and JNK/SAMP activation. The activated p38 MAPK and JNK/SAPK promotes transcriptional activation of p53 and FoxO1 by acetylation. P53 and FoxO1-mediated up-regulation of p16^INK4A^ and p21^CIP1^ distrubs cyclins and CDKs, which increase protein stability of pRB and allow to G_0_/G_1_ cell-cycle arrest and senescence. Conversely, activated p38 MAPK and JNK/SAPK reduce SIRT-1 level by phosphorylating Ser47, eventually losing deacetylation activity. However, treatment with antcin M activates Nrf2-dependent anti-oxidant genes such as HO-1 and NQO-1 followed by activation of PI3K/AKT and ER1/2 kinases, which facilitates ROS inhibition and upregulates SIRT-1 expression in HNDFs and HUVECs. Results expressed as mean ± SEM of three indipendent expriments. Statistical significance at ^Ф^*P* < 0.05 compared to NG *vs.* HG and *P < 0.05 compared to HG vs. samples.

Next to investigate whether the protective effect of ANM is limited to fibroblasts or extends to other organs, we examined hyperglycemia-induced endothelial senescence and the protective effect of ANM. Interestingly, ANM showed a similar protective effect against HG-induced endothelial senescence. Taken together, data from the current study thus support the hypothesis that ANM promotes anti-oxidant defense and SIRT-1 stability in hyperglycemia-induced dermal fibroblasts and endothelial cells that minimize cellular senescence and growth arrest (Figure 10). Further *in vivo* studies demonstrate that ANM is a novel anti-aging reagent that conferred an increase in oxidative stress resistance and extends life span on the nematode *C. elegans*.

## MATERIALS AND METHODS

### Chemicals and reagents

Antcin A, antcin B, antcin C, antcin H, antcin K and antcin M were isolated from the fruiting bodies of *A. cinnamomea* and *A. salmonea* as described previously [17]. The purity of the antcins was above 99% as confirmed by HPLC and FT-NMR analysis. Minimum essential medium (MEM), Medium 199 (M-199), fetal bovine serum (FBS), sodium pyruvate, penicillin and streptomycin were obtained from Invitrogen (Carlsbad, CA). Heparin sodium salt, endothelial cell growth supplement (ECGS), *N*-acetylcysteine, 2′, 7′-dichlorofluorescein diacetate (DCFH_2_-DA), 3-(4,5-dimethyl-thiazol-2-yl)-2,5-diphenyl tetrazolium bromide (MTT) and D-Glucose were purchased from Sigma-Aldrich (St Louis, CA). Antibodies against cyclin D1, cyclin B1, cyclin E, CDK2, CDK4, CDK6, Cdc2, phos-pRb, p16^INK4A^, p21^CIP1^, acety-p52, phos-p53, phos-FoxO1, FoxO1, phos-JNK/SAPK, JNK/SAPK, phos-p38 MAPK, p38 MAPK, phos-ERK1/2, ERK1/2, Phos-AKT, AKT, histone H3, phos-SIRT-1, SIRT-1, SIRT-3, SIRT-6 and Keap-1 were obtained from Cell Signaling Technology, Danvers, MA. Antibodies against p53, SMP30 and acetyl-FoxO1 were purchased from Santa Cruz Biotechnology, Dallas, TX. Antibodies against HO-1 and NQO-1 were obtained from Abcam, Cambridge, UK. All other chemicals were reagent grade or HPLC grade and supplied by either Merck (Darmstadt, Germany) or Sigma-Aldrich.

### Cell culture and sample treatment

Human normal dermal fibroblasts (HNDFs, CCD966SK) and human umbilical vein endothelial cells (HUVECs) were obtained from the Bioresource Collection and Research Center (BCRC), Hsinchu, Taiwan. HNDFs were grown in MEM containing 10% FBS, 2 mM L-glutamine, 100 U/mL penicillin and streptomycin at 37°C in a fully humidified atmosphere of 5% CO_2_. Likewise, HUVECs were grown in M-199 medium supplemented with ECGS, heparin, 10% FBS and 100 U/mL penicillin and streptomycin at 37°C in a fully humidified atmosphere of 5% CO_2_. High-glucose treatment was performed by treating cells with 15 or 30 mM D-glucose (HG) for 24-72 h. HNDFs and HUVECs were also treated with HG in the presence of 10 μM ANM or 100 μM *N*-acetylcysteine or 5 μM resveratrol. Controls were performed in the presence of media with normal glucose alone (NG, 5.5 mM) or with 10 μM ANM or 100 μM *N*-acetylcysteine or 5 μM resveratrol.

### Cell viability and proliferation assay

Cell viability was assessed by MTT colorimetric assay. Briefly, HNDFs (2 × 10^4^ cells/well) or HUVECs (5 × 10^4^ cells/well) were seeded in a 24-well culture plate. After treatment with HG (15 and 30 mM) in the presence or absence of samples for 24-72 h, culture media was withdrawn and incubated with MTT (1 mg/mL) in fresh medium for 2 h. The MTT formazan crystals were dissolved in 400 μL of DMSO and the samples were measured at 570 nm (A_540_) using an ELISA microplate reader (Bio-Tek Instruments, Winooski, VT). The percentage of cell viability (%) was calculated as (A_570_ of treated cells/A_570_ of untreated cells) × 100.

Cell proliferation was evaluated using trypan blue exclusion assay as described previously [49] with minor modification. Cells were plated into 6-well plates at a density of 5 × 10^4^ cells/well. After incubation overnight, cells were treated with test samples in the presence or absence of HG for 24-72 h. Cells exposed to 0.2% Trypan blue were then counted in a hemocytometer, and cells stained with Trypan blue were excluded. Percentage of viable cells was calculated based on the ratio of viable cells to total cell population in each well. The proliferation rate was calculated based on the number of viable cells in HG or sample-treated groups *versus* the NG-treated group.

### Apoptosis assay

The assay of Annexin V and PI binding staining was performed with an Annexin V-FITC/PI Apoptosis Detection Kit according to the manufacturer's instructions (BD Bioscences, San Jose, CA). Briefly, 5 × 10^5^ cells/dish were seeded in a 10 cm culture dish, after incubation overnight, cells were exposed to HG (15-30 mM) or NG (5.5 mM) for 72 h. Cells were washed twice with PBS and collected using 0.25% trypsin without EDTA, cells were pooled by centrifuging at 1500 × *g* for 5 min. Then, cells were suspended in 500 μL of binding buffer which contained 1 μL Annexin V-FITC and 5 μL PI and incubated with the cells for 5 min in the dark. The stained cells were analyzed directly by flow cytometer (Beckman Coulter, Brea, CA). Data were acquired and analyzed using CXP software (Beckman Coulter).

### Cell-cycle analysis

HNDFs at a density of 5 × 10^5^ cells in 10 cm dishes were treated with ANM or NAC in the presence or absence of HG for 72 h. Cells were collected, washed with PBS and fixed in 95% cold-ethanol, and kept at −20°C overnight. The cell pellet was then washed again with PBS and centrifuged at 1500 × *g* for 5 min. The pellet was re-suspended in 1 mL PI/Triton X-100 (20 μg/mL PI, 0.1% Triton X-100 and 0.2 mg/mL RNAse) and incubated on ice for 30 min. The total cellular DNA content was analyzed with a flow cytometer (Beckman Coulter FC500). Data were acquired and analyzed using CXP software (Beckman Coulter).

### Flow cytometric detection of intracellular ROS

Intracellular ROS accumulation was determined using the dye DCFH_2_-DA following a procedure described earlier [50]. Briefly, HNDFs (1 × 10^5^ cells/well) were seeded in 6-well plates and incubated with HG in the presence or absence of test samples for 24 h. At the end of the incubation, the culture supernatant was removed and cells were washed twice with PBS. DCFH_2_-DA (10 μM) was mixed with 500 μL MEM and added to the culture plate. After incubation for 30 minutes, cells were collected by trypsin and the fluorescence shift was quantified using a flow cytometer (Beckman Coulter). Data were acquired and analyzed using CXP software (Beckman Coulter).

### Senescence-associated β-galactosidase activity assay

Senescence-associated β-galactosidase (SA-β-gal) activity was determined in formaldehyde-fixed histochemical staining kit according to the manufacturer's instructions (Cell Signaling Technology, Danvers, CA). Briefly, cells were grown in 6-well plates at a density of 5 × 10^4^ cells/well, and incubated with HG or test samples for 48 h (HUVECs) or 72 h (HNDFs). After incubation, cells were stained with SA-β-gal staining solution at pH 6.0 overnight and then the development of blue staining was observed and photographed under a bright-field microscope (Motic Electric Group, Xiamen, P.R. China).

### Immunofluorescence

HNDFs at a density of 1 × 10^4^ cells/well were cultured in an eight-well glass Nunc Lab-Tek chamber (ThermoFisher Scientific, Waltham, MA). Cells were treated with ANM or NAC in the presence or absence of HG for 2-72 h. After incubation, culture medium was removed and cells were fixed in 4% paraformaldehyde for 15 min, permeabilized with 0.1% Triton X-100 for 10 min, washed and blocked with 10% FBS in PBS, and then incubated overnight with the corresponding primary antibodies in 1.5% FBS. The cells were then incubated with the fluorescein isothiocyanate (FITC)-conjugated secondary antibody (Alexa fluor 488, ThermoFisher Scientific) for another 1 h in 6% bovine serum albumin (BSA). Next, the cells were stained with 1 μg/mL 4′,6-diamidino-2-phenylindole (DAPI, Cell Signaling Technology) for 5 min, washed with PBS, and visualized using a fluorescence microscope (Motic Electric Group) at 40 × magnification.

### RNA extraction and Q-PCR analysis

Total RNA was extracted from cultured HNDFs using Trizol Reagent (Thermo Fisher Scientific). Q-PCR analysis was performed using Applied Biosystems detection instruments and software. Forward and reverse primers (10 μM), and the working solution SYBR green, was used as a PCR master mix, under the following conditions: 96°C for 3 minutes followed by 40 cycles at 96°C for 1 minute, 50°C for 30 seconds and 72°C for 90 seconds. GAPDH was used as an internal standard to control for variability in amplification because of differences in starting mRNA concentrations. The copy number of each transcript was calculated as the relative copy number normalized by GAPDH copy number. The sequences of the PCR primers were as summarized in Table 1.

**Table 1:** Oligonucleotides used for Q-PCR

Gene	Sequence	Reference
SIRT-1	Forward: 5′-GCAGATTAGTAGGCGGCTTG Reverse: 5′-TCTGGCATGTCCCACTATCA	[28]
SIRT-3	Forward: 5′-CATGAGCTGCAGTGACTGGT Reverse: 5′-GAGCTTGCCGTTCAACTAGG	[28]
SIRT-6	Forward: 5′-AGGATGTCGGTGAATTACGC Reverse: 5′-AAAGGTGGTGTCGAACTTGG	[28]
HO-1	Forward: 5′-TCAACGGCACAGTCAAGG-3′ Reverse: 5′-ACTCCACGACANACTCAGC-3′	[19]
NQO-1	Forward: 5′-TGCGGTGCAGCTCTTCTG-3′ Reverse: 5′-GCAACCCGACAGCATGC-3′	[19]
β-actin	Forward: 5′-TCAACGGCACAGTCAAGG-3′ Reverse: 5′-ACTCCACGACANACTCAGC-3′	[19]

### Protein extraction and western blot analysis

HNDFs or HUVECs (1 × 10^6^ cells/dish) were cultured in 10-cm dishes and treated with ANM or NAC or RES in the presence or absence of HG for 48-72 h. Cells were lysed by either RIPA lysis buffer or nuclear and cytoplasmic extraction reagents (Thermo Fisher Scientific). Protein concentrations were determined by Bio-Rad protein assay reagent (Bio-Rad Laboratories, Hercules, CA). Equal amounts of protein samples (60 μg) were separated by 7-12% SDS-PAGE and the separated proteins were transferred onto polyvinylidene chloride (PVDC) membrane overnight. The transferred protein membranes were blocked with 5% non-fat dried milk for 30 min, followed by incubation with specific primary antibodies overnight, and either horseradish peroxidase-conjugated goat anti-rabbit or anti-mouse antibodies for 2 h. The blots were detected using VL Chemi-Smart 3000 (Viogene Biotek, Sunnyvale, CA) with the enhanced chemiluminescence (ECL) western blotting reagent (Millipore, Billerica, MA).

### Immunoprecipitation

HNDFs were seeded at a density of 1 × 10^6^ cells/dish in 10 cm dish and treated with ANM or pharmacological inhibitors of AKT, JNK/SAPK, p38 MAPK and ERK1/2 in the presence or absence of HG. After treatment, cells were lysed with RIPA buffer containing protease inhibitor cocktail. The lysates were homogenized and centrifuged at 16,000 × *g* for 15 min at 4°C. The supernatant was collected and the protein concentration was determined by Bio-Rad protein assay reagent. Total protein extract containing 500 μg of proteins were precleared with protein-A agarose beads for 1 h and incubated with 3 μg of anti-Keap-1 antibody for overnight at 4°C with gently shake. After overnight incubation, centrifuged at 2000 × *g* for 5 min at 4°C, the supernatant was discarded and the reaming pellet was washed with RIPA buffer. Immunoprecipitated complexes were mixed with SDS sample buffer and denatured at 94°C for 5 min. Equal amount of protein samples were subjected to western blotting. The ubiquitinated Keap-1 protein levels were determined by ubiquitin antibody.

### Gene silencing by siRNA

HNDFs (2.5 × 10^5^ cells/dish) were cultured in 6 cm dishes, after 60% confluence at the time of transfection, culture media was replaced with 2 mL of Opti-MEM (Invitrogen) and cells were transfected using Lipofectamine RNAiMax (Invitrogen) transfection reagent. For each transfection, 5 μL of RNAiMAX was mixed with 500 μL of Opti-MEM and incubated for 5 min at room temperature. In a separate tube, siRNA (100 pM for a final concentration of 100 nM in 1 mL Opti-MEM) was added to 500 μL of Opti-MEM and the siRNA solution was added to the diluted RNAiMAX reagent. The resulting siRNA/RNAiMAX mixture (1 mL) was incubated for an additional 25 min at room temperature to allow complex formation. Subsequently, the solution was added to the cells in the 6-well plates, giving a final transfection volume of 2 mL. After 6 h incubation, the transfection medium was replaced with 3 mL of standard growth medium and the cells were cultured at 37°C. After transfection for 24 h, cells were treated with ANM, NAC or RES in the presence or absence of HG, and subjected to subsequent experiments.

### Luciferase reporter assay

ARE promoter activity was measured using a dual-luciferase reporter assay system (Promega, Madison, WI). Briefly, HNDFs or HUVECs (1 × 10^5^ cells/well) were cultured in 6-well plates until ~80% confluence and then incubated for 5 h in Opti-MEM that did not contain antibiotics. Cells were then transfected with ARE plasmid (Qiagen, Hilden, Germany) using Lipofectamine 2000 (Invitrogen) and incubated for 36 h. After plasmid transfection, cells were treated with ANM (10 μM) or NAC (100 μM) or RES (5 μM) in the presence or absence of HG (30 mM) for 6 h. The cell lysate was prepared and incubated with luciferase agents and the relative luminescence intensity was quantified using a spectrophotometer (Hidex Oy, Turku, Finland).

### *C. elegans* strain

The wild type Bristol N2 strain was used in this study. *C. elegans* and *Escherichia coli* OP50 strain were obtained from the Caenorhabditis Genetic Center, University of Minnesota (Minneapolis-St. Paul, MN). Worms were maintained at 20°C on nematode growth medium (NGM). Hatched worms (L1-stage larvae) were transferred to fresh agar plates and cultured with *E. coli* OP50 as a food source until they reached the L4 larvae stage. Synchronization of worm cultures was achieved by hypochlorite treatment of gravid hermaphrodites.

### Stress-resistance assay

Age synchronized L1 larvae were incubated with liquid S-basal medium containing E. coli OP50 at a density of 1 × 109 cells. Ml and 10 and 20 μM ANM or 0.01% DMSO (vehicle control) for 3 days. Subsequently, adult worms were subjected to oxidative stress assay. To induce oxidative stress, worms were incubated with Juglone (5-hydroxyl-1,4-naphthoquinone; Sigma), an ROS-generating agent. ANM treated and control worms were transferred to S-basal medium containing 250 μM Juglone, and incubated from 2.5, 3.5 and 4.5 h. After treatment, viable worms were scored. Worms were scored as dead when they failed to response physical touch. The test was performed triplicate.

### Hyperglycemia-induced life span assay

For the life span assay, age synchronized L1 larvae were transferred to NGM plates containing ANM (10 μM) or RES (438 μM) with or without high-glucose (50 mM). Control worms were treated with 0.1% DMSO. All worms were kept 20°C to develop adulthood. Surviving and dead animals were counted daily (starting from the first day of adulthood) until all worms had died. Animals that did not move when gently prodded were scored as dead. Worms suffering from internal hatch (a defect in egg-laying) and those that crawled off the NGM plate were not included in the life-span assay. During the reproductive period, adult worms were transferred to fresh NGM plates every and then every other day thereafter. Life span assay result was obtained from three independent assays.

### Statistical data analysis

Data are expressed as mean ± S.E.M. All data were analyzed using the statistical software Graphpad Prism version 6.0 for windows (GraphPad Software, La Jolla, CA). Statistical analysis was performed usingone-way ANOVA followed by Dunnett's multiple comparisons test with a P value of less than 0.05 indicating statistical significance.

### Highlights

1. Antcin M protects dermal fibroblasts from hyperglycemia-induced cell-cycle arrest.
2. Antcin M protects dermal fibroblasts from hyperglycemia-induced oxidative injury.
3. Antcin M activates Nrf2-mediated antioxidant genes in dermal fibroblasts.
4. Antcin M upregulates SIRT-1 expression in dermal fibroblasts.
5. Antcin M protects and extends *C. elegans* life span under stress condition.

## Abbreviations

ANA: antcin A
ANC: antcin C
ANH: antcin H
ANK: antcin K
ANM: antcin M
CDK: cyclin-dependent kinase
DAPI: 4′,6-diamidino-2-phenylindole
DCFH-DA: 2′, 7′-dichlorofluorescein diacetate
FBS: fetal bovine serum
HG: high-glucose
HNDFs: human normal dermal fibroblasts
HO-1: hemoxygense-1
HUVECs: human umbilical vein endothelial cells
MEM: minimum essential medium
MTT: 3-(4,5-dimethyl-thiazol-2-yl)-2,5-diphenyl tetrazolium bromide
NAC: *N*-acetylcysteine
NG: normal-glucose
NQO-1: NAD(P)H dehydorogenase (quinone 1)
Nrf2: Nuclear factor (erythroid-derived 2)-like 2
pRb: protein retinoblastoma
RES: resveratrol
ROS: reactive oxygen species
SA-β-gal: senescence-associated-β-galactosidase
SIPS: stress-induced premature senescence
SIRT-1: silent mating type information regulation 2 homolog
SMP30: senescence-associated marker protein 30
